# Who says “no” to participating in stroke clinical trials and why: an observational study from the Vancouver Stroke Program

**DOI:** 10.1186/s13063-019-3434-0

**Published:** 2019-05-31

**Authors:** Zoe R. O’Neill, Halina M. Deptuck, Lauren Quong, Genoveva Maclean, Karina Villaluna, Princess King-Azote, Mukul Sharma, Ken Butcher, Robert G. Hart, Thalia S. Field

**Affiliations:** 1 Vancouver Stroke Program - Research Office, 8295–2775 Laurel Street, Vancouver,, BC V5Z 1M9 Canada; 2 Population Health Research Institute, Hamilton Health Sciences, 237 Barton Street East, Hamilton, ON L8L 2X2 Canada; 3 University of New South Wales, Prince of Wales Clinical School, Level 1, South Wing, Edmund Blacket Building Prince of Wales Hospital, Ranwick, NSW 2031 Australia; 4 Faculty of Medicine, University of British Columbia, S169-2211 Wesbrook Mall, Vancouver, BC V6T 2B5 Canada; 5 Faculty of Medicine, Memorial University, 300 Prince Philip Drive, St. John’s, NL A1B 3V6 Canada; 6 Faculty of Medicine, McMaster University, 237 Barton St. E., Hamilton, ON L8L 2X2 Canada

**Keywords:** Clinical trials, Enrolment, Refusals, Recruitment, Gender

## Abstract

**Background:**

Successful stroke trials require adequate recruitment. In this observational study, we assessed reasons for refusal to provide informed consent in eligible patients approached for clinical trial participation at the Vancouver Stroke Program.

**Methods:**

We assessed screening logs from four trials that were actively recruiting at our center: three randomized trials, two of which investigated different antithrombotic strategies for secondary prevention (NAVIGATE-ESUS, NCT02313909 12/2014; DATAS-II, NCT02295826 11/2014) and one that investigated surgery plus medical management versus medical management alone for primary prevention (CREST-2, NCT02089217 03/2014). The fourth study was observational and non-randomized; all participants received an external monitoring device (PROPHECY, NCT03712865 10/2018). Screening logs from June 2015 to April 2017 were reviewed retrospectively. Subsequently, we used a prospective structured case report form for screening (May 2017–March 2018). We assessed and compared refusal rates between trials, demographics of those refusing consent, and their reasons for doing so. We used descriptive statistics, chi-square and Fisher’s exact tests as appropriate for non-parametric data, and t-tests for parametric data. We examined likelihood of refusal by sex using multivariable logistic regression models including age and trial intervention as co-variables.

**Results:**

A total of 235 patients (43% women) were approached for consent. More patients refused the surgical (59%) and antithrombotic trials (53%) compared with the non-randomized external monitoring device study (13%) (*p* < 0.001). Surgical trial refusals were primarily due to a desire for certainty in receiving a particular intervention (39%), with the majority of those patients wanting surgery. Refusals for the antithrombotic trials were mainly due to concerns with the potential side effects of the study drug (41%); refusals in the device trial were mainly due to disinterest (46%). Women refused participation more often than men (48% vs 33%). Women remained less likely to consent than men, even after adjustment for age and trial intervention (OR 0.46, 95% CI 0.26–0.82, *p* = 0.009).

**Conclusions:**

Concern surrounding drug safety, randomization, and disinterest were the chief deterrents to enrolment; there were also differences in rates of consent by gender. A better understanding of why patients refuse participation in stroke trials may help to develop future patient-directed communication strategies to improve enrolment. Further research is required to better understand the reasons underlying gender disparities in consent rates.

**Electronic supplementary material:**

The online version of this article (10.1186/s13063-019-3434-0) contains supplementary material, which is available to authorized users.

## Background

Reliable recruitment is crucial for the success of a clinical trial. Failure to recruit is a serious methodological concern [1], as inadequate enrolment can lead to premature trial termination, suboptimal use of resources, underpowered results [2], and risk of non-publication [3].

There is a shortage of refusal data for stroke clinical trials; further details may help to improve recruitment strategies and thus enhance clinical trial efficiency. Commonly cited decision factors among eligible candidates for cardiovascular clinical trials include uneasiness regarding the randomization process and not knowing what treatment would be assigned, the concept of participating as a test subject (i.e. perceiving that he/she is a “guinea pig”), fear of potential side effects of study medications, and the treating physician’s attitude towards the trial [4]. Patients also report that the demands necessitated by a study, such as additional procedures and appointments, deter them from participating. Finally, preference for a particular treatment or uncertainty about study treatments are also barriers [5].

Identifying barriers to recruitment may facilitate targeted strategies in order to improve study participation and increased clinical trial efficiency. Our aim was to explore demographic characteristics of eligible clinical trial participants providing and refusing informed consent and to identify reasons for clinical trial refusals. We hope to add to the literature more detailed information that may be used to improve recruitment, and hence efficiency, of clinical trials in stroke. Further, most clinical trials in stroke have a minority of female participants [6] and we wanted to explore whether consent rates by gender may contribute to this imbalance.

## Methods

### Data collection

This was a single-center study. We initially used retrospective data from screening logs at the Vancouver Stroke Program where reasons for refusal were routinely recorded from four actively recruiting clinical trials: one non-randomized device trial for detection of atrial fibrillation; one randomized surgical trial for primary prevention; and two randomized antithrombotic trials for secondary stroke prevention (Table 1; inclusion/exclusion criteria summarized in Additional file 1: Table S1). These studies were selected because there was adequate detail in the screening logs to ascertain patient age, sex, and reason for refusal. Of eight ongoing trials at our center, two examined acute antithrombotic interventions, one investigated an acute hemostatic therapy, and one observational diagnostic study did not have enough information in the screening logs for inclusion in this study.

**Table 1:** Characteristics of the clinical trials being assessed for participation rates

Intervention	Antithrombotic agent (#1)	Antithrombotic agent (#2)	Surgery	Device
Trial design	Randomized	Randomized	Randomized	Non-randomized
Enrolment time window	< 72 h from symptom onset	< 6 months from symptom onset	< 6 months from diagnosis asymptomatic	< 6 months from symptom onset
Brief description of study	Novel anticoagulant vs standard of care for secondary prevention of ischemic stroke	Novel anticoagulant vs standard of care for secondary prevention of ischemic stroke	Surgery plus medical management vs medical management alone for primary prevention of ischemic stroke	30-day external loop recorder to look for atrial fibrillation

We also collected data prospectively using a structured case report form including age, sex, date of qualifying event, date of approach for participation, and a categorized list of reasons for refusal derived from our retrospective findings [7, 8], with room for free text if required. When individuals were approached, those who refused consent were asked about their reasons for doing so in an open-ended fashion. Categorization of reasons for refusal were adjudicated by two research assistants (LQ and ZO) and reviewed by a senior author (TF) for consensus.

### Study population and setting

Potential participants were recruited from the Emergency Department, stroke unit, or outpatient stroke prevention clinic. Patients were approached if they met study-specific eligibility criteria for the respective clinical trial as per screening clinician or research coordinator. We included all patients being approached for consent to participate in any of the four trials. Three of the four trials included in this study recruited from an outpatient stroke prevention clinic. The acute antithrombotic trial (#1) recruited candidates from the Emergency Department of the hospital stroke ward. From June 2015 to April 2017, data are retrospective. To ensure that there was no bias in data collection from the retrospective screening logs, which are generally known to be incomplete [9], data were then collected prospectively from May 2017 to March 2018 using a structured case report form.

This study was a quality improvement initiative for the Vancouver Stroke Program and thus informed consent from study participants for data collection was not required during either the prospective or retrospective periods as per local protocols. We collected no personally identifying information from participants and subsequently applied to our University Clinical Research Ethics Board for approval to submit this manuscript for publication.

### Statistical methods

Data were analyzed with descriptive statistics (counts and percentages). When comparing groups, we used a two-sample test with continuity correction for parametric data and Pearson’s Chi-square and Fisher’s exact tests as appropriate for non-parametric data. After inspecting demographic characteristics between participants in the retrospective and prospective epochs (Table 2), we felt that these groups were similar enough to combine for the main analysis. We explored likelihood of refusal by gender with a multivariable logistic regression model, adjusting for age and trial intervention and examining for an age*gender interaction. Age data were missing for two refusal participants in the retrospective data and two refusals in the prospective data. Sex was missing for one refusal participant in the prospective data. Missing data were treated with pairwise deletion. All analyses were performed with SPSS Version 25 (Armonk, NY, USA).

**Table 2:** Demographic information of patients approached for clinical trial participation in the retrospective and prospective epochs

	Retrospective	Prospective	*p* value
Total approached (*n*)	132	103	–
Refusal Rate (*n* (%))	51 (39)	42 (41)	0.74^a^
Mean age (years ± SD)	69.4 ± 10.3	72.9 ± 8.9	0.007^b^
Male (*n* (%))	81 (61)	51 53 (52)	0.15^a^

^a^Chi-square

^b^T-test

## Results

During the study, 235 candidates (132 retrospective, 103 prospective) were approached. Men comprised a larger proportion of those approached (57%). Mean age was 70.9 years (SD 9.85), with male candidates being younger (68.2 men vs 74.6 women, *p* < 0.001). There was no significant difference in the mean age of those refusing versus those consenting to trial participation (70.7 ± 9.7 vs 71.1 ± 10.0, *p* = 0.76). Patients in the prospective cohort were slightly older (69.4 ± 10.3 vs 72.9 ± 8.9, *p* = 0.007).

Of all candidates approached, 40% refused enrolment (Table 3). The proportion of candidates refusing participation differed between trial intervention (antithrombotic 53%, device 15%, and surgery 59%, *p* < 0.001). The device trial had the lowest refusal rate (13%). There was no difference in proportion of refusals for the antithrombotic trial recruiting from acute care (47% of 53 patients) versus the other trials which recruited from the outpatient clinic (37% of 182, *p* = 0.20). Rates of refusal between the two antithrombotic trials, which differed in their time window for enrolment (72 h and recruiting from acute care versus six months after stroke/TIA and recruiting from the outpatient clinic), were similar (47% vs 59%, *p* = 0.31). There was also no difference in likelihood of refusal based on location/acuity for the trials overall (crude odds ratio [OR] 1.50, 95% confidence interval [CI] 0.81–2.78, *p* = 0.20), adjusted OR including gender, age, intervention in multivariable logistic regression model 1.43 (95% CI 0.68–2.99, *p* = 0.35) (Additional file 1: Table S2).

**Table 3:** Refusal rates overall and by trial intervention

	Antithrombotic agent	Surgery	Device	Overall
Consented (*n* (%))	52 (47)	16 (41)	74 (87)	142 (60)
Refused (*n* (%))	59 (53)	23 (59)	11 (13)	93 (40)
Approached (*n)*	111	39	85	235

Reasons for refusal are summarized in Table 4. Concern about the risks of the study drug was the primary reason for refusal in the antithrombotic trials (41%). Candidates often described that they did not feel comfortable being on a “stronger blood thinner” and wanted to stay on “traditional” or “standard of care” medications. Disinterest in participating in the study (15%) and inability to commit to the study follow-up schedule (10%) were also common reasons for refusal in the antithrombotic trials. Discomfort with randomization (39%) was the chief reason for refusal in the surgical intervention trial, followed by disinterest (30%) and inability to adhere to follow-up (17%). In the device trial, disinterest (46%) was the primary reason for refusal, followed by concerns about using the study monitoring device (18%) and issues with follow-up (9%). Concerns about the ability to attend follow-up study visits were mostly (50%) a result of participants living outside of the city of Vancouver. Of those that described being “not interested” as a reason for refusal, several explained that the study follow-up period was too long and a big commitment. Additionally, 3/21 who were “not interested” had the study information explained to them second-hand through a translator.

**Table 4:** Summary of reasons for refusal across all recruiting trials, in absolute numbers and as a percentage of total refusals (*n* = 93)

Reason	Antithrombotic	Surgical	Device	*n* (%)
Concern about receiving study drug/device	24	0	2	26 (28)
Not interested in participating in study	9	7	5	21 (23)
Concern about inability to choose intervention vs control	3	9	0	12 (13)
Unable to commit to follow-up appointments	6	4	1	11 (12)
Too stressed or busy	2	1	1	4 (4)
Concerns regarding eligibility for travel insurance	3	0	0	3 (3)
Indecision about participation	2	0	0	2 (2)
Family refusal on patient’s behalf	2	0	0	2 (2)
Multiple medical comorbidities	0	1	0	1 (1)
In denial about medical condition	1	0	0	1 (1)
Unknown	7	1	2	10 (11)
Total	59	23	11	93 (100)

Men were less likely to refuse participation (33%) than women (48%, *p* = 0.02). Women remained less likely to consent than men, even after adjustment for age and trial intervention (adjusted OR 0.46, 95% CI 0.26–0.82, *p* = 0.009) (Table 5). There was no significant interaction between age and gender. Reasons for refusal were similar between genders. Men tended to be less interested in participating in studies (27% vs 19%) and women expressed concern about study drug more frequently (33% vs 21%) (Additional file 1: Table S3).

**Table 5:** Multivariable logistic regression, odds of consenting to trial participation

Odds ratio (95% CI), *p*-value		
Model 1 (gender only)	Model 2 (+age)	Model 3 (+age, intervention)
0.53 (0.31–0.90), p = 0.02	0.46 (0.26–0.82), *p* = 0.008	0.46 (0.26–0.82), *p* = 0.009

## Discussion

We found that the device trial had the lowest rate of refusal, followed by the antithrombotic and surgical intervention trials. In the antithrombotic trial, concern about randomization to the study drug was the most commonly cited reason for refusal. In the surgical trial, concern about randomization precluding the ability to choose an intervention was the principal reason for refusal. A disinterest in participating in the study was the most common reason for refusal in the device trial. Additionally, we found that women were more likely to refuse participation than men. We found no difference in refusal rates between the two antithrombotic trials, despite there being a difference in the acuity of the enrolment window between the two studies, or between the trial recruiting from the acute care setting versus the outpatient setting.

Patients are more likely to refuse participation in a clinical trial when they perceive an increased risk to their own health [10]. The lower rates of refusal seen in the device trial could be attributed to a greater aversion to the risks associated with other study interventions. Namely, even if the absolute risk of rash were higher than the risks of serious adverse events from a surgical or antithrombotic intervention, the risk of skin rash from the electrode pads in the device trial would be seen as less hazardous than an elective surgery with associated perioperative risks or antithrombotic medication with risk of bleeding.

In patients approached for antithrombotic trials, there was particular concern over randomization to the study drug, which was associated with an increased bleeding risk compared to the control therapy. Patients may be more hesitant to participate in a trial when there is a chance of being randomized to a higher-risk intervention. Some patients prefer the certainty of knowing which therapy they will receive instead of leaving the decision to chance. In the surgical intervention trial, patients often expressed the desire for autonomy to choose between the study treatment arms. This discomfort with randomization has been confirmed in previous surgical intervention studies [11]. Consistent with other work [1, 12], our analysis found that disinterest was a common reason for participation refusal, particularly in the device study. Previous work investigating home health monitoring devices suggests that patients often have no interest in using a device or they perceive it as unnecessary [13]. Strategies that stimulate patient interest in studies may be important to augment recruitment.

There is a lack of consensus about whether clinical trial participation differs by gender. Here, we report that women show a greater likelihood of refusing participation in studies and that this difference occurred independently of age or trial intervention. However, some studies have found the opposite, while others found no differences between male and female participation [14]. Women are consistently reported as underrepresented in stroke clinical trials, often accounting for < 30% of participants in stroke trials [15]. There are several proposed reasons for this disparity including clinical trial designs where studies recruit an exclusively male cohort [6]. Additionally, since women are generally older than men at time of stroke, they may have more medical co-morbidities that act as exclusion criteria [16]. Other literature suggests that women may be more risk-averse and uncomfortable with the uncertainty that comes from participating in randomized controlled trials [17]. Although there was no significant difference in proportions by gender in the retrospective and prospective period, there was a greater trend towards gender parity in the prospective period. It is possible that this difference is a product of unconscious monitoring of research approaches by gender as a result of the sex discrepancy found in the retrospective review of screening logs, which acted as a corrective action to ensure equal approaches to men and women.

Previous research suggests that patients are more likely to consent to trials if they have been given sufficient time to decide [18]. Use of a legally authorized representative in an acute setting may also affect the consent process and outcome. Our acute antithrombotic trial enrolled patients with minor stroke/TIA who were able to provide their own consent. We did not find a difference in refusal rates between the two antithrombotic trials, despite different recruitment windows and settings. We did not record the amount of time that individual patients were given to decide about participating in a given trial, but this may affect participation rates and should be considered in future work.

Physician attitudes also influence a patient’s decision to participate in a research trial [19]. In the case of a randomized trial, the perception that one’s physician may be biased towards a particular intervention may impact willingness to provide informed consent. Alternatively, a patient may have the impression that their physician feels that a particular trial is not important or an inconvenience and may refuse consent for those reasons.

In order to overcome barriers to patient recruitment in stroke clinical trials, interventions that improve patient education about the research process may be helpful. A better understanding of the importance of randomization for valid study results may encourage less skepticism on the part of the patient. Patients are becoming increasingly empowered and involved in treatment decisions. Research studies and consent processes must be designed to align with this reality.

This study has limitations. Patient medical history was not collected in the screening logs and the study population was a sample of convenience given that it was limited to trials with the most detailed screening logs. Furthermore, the retrospective data were collected from research coordinator notes and recall of events. The lack of a structured data collection form during the retrospective period creates additional room for bias in the reported reasons for refusal [9]. However, it is reassuring that there were no major demographic differences or differences in rates or reasons for refusal between the retrospective and prospective groups. Although patients in the prospective group were statistically significantly older (69 years vs 73 years), participation rates and gender ratios between the retrospective and prospective datasets were similar, as were reasons for refusal. Our multivariable analysis did not find any significant effect of age on consent, either in the combined or separated cohorts, nor was there an age-gender interaction.

The non-randomized nature of the study may have introduced implicit bias when recruiting and approaching patients for research participation. This may have prevented research staff from approaching patients from certain demographic populations, such as homeless individuals [20], individuals with a history of addiction, patient with seemingly complicated family dynamics, and patients in whom there was a language barrier.

## Conclusions

Our analysis revealed that therapeutic intervention trials had the highest rates of refusal and that women refused study participation more often than men. The most common reason for refusal was concern about receiving the study drug, followed by lack of interest in participating in the research study. In planning future clinical trials, the informed consent process should incorporate strategies to surmount potential barriers to participation, including gender-specific issues and education around the randomization process.

## Additional file

Additional file 1: **Table S1** Summary of eligibility criteria for trials included in analysis. **Table S2.** Odds of refusal rate by non-acute/outpatient vs acute/emergency department, multivariable logistic regression. **Table S3.** Reasons for refusal separated by gender. (DOCX 24 kb)

## Data Availability

The datasets used and analyzed during the current study are available from the corresponding author on reasonable request.

## Abbreviations

ESUS: Embolic Stroke of Undetermined Source
TIA: Transient ischemic attack
