# Supplementary material for: Who says “no” to participating in stroke clinical trials and why: an observational study from the Vancouver Stroke Program
Source: Trials. 2019 May 31;20:313. doi: 10.1186/s13063-019-3434-0 (PMC6545028; doi:10.1186/s13063-019-3434-0)
Supplement: Supplementary file 1 — Table S1 Summary of eligibility criteria for trials included in analysis. Table S2. Odds of refusal rate by non-acute/outpatient vs acute/emergency department, multivariable logistic regression. Table S3. Reasons for refusal separated by gender. (DOCX 24 kb) [file 13063_2019_3434_MOESM1_ESM.docx]

**SUPPLEMENTARY APPENDIX**

**Table S1.** Summary of eligibility criteria for trials included in analysis

| **Intervention** | **Eligibility Criteria** |
| --- | --- |
| **Antithrombotic agent (1)** | - > 18 years of age - TIA or minor stroke (NIHSS <9) - Symptom onset is < 72 hours prior to enrollment - DWI lesions volume < 25 mL - Patients without DWI lesions can be included if clinical history consistent with TIA |
| **Antithrombotic agent (2)** | - ≥ 50 years of age - Recent (between 7 days and 6 months) embolic stroke of uncertain source defined as: - Recent ischemic visualized by brain imaging that is not lacunar - Absence of cervical carotid stenosis > 50% - No atrial fibrillation - No intra-cardiac thrombus - No other specific cause of stroke |
| **Surgery** | - ≥ 50 years of age - Carotid stenosis ≥ 70% - No medical history of stroke or TIA ipsilateral to stenosis within 180 days of randomization - mRS 0-1 - Carotid stenosis must be treatable with CEA or CAS |
| **Device** | - ≥ 55 years of age - Diagnosis of embolic stroke or TIA of uncertain source occurring within previous 6 months - No previous ECG or holter monitor showing atrial fibrillation or atrial flutter - Patient has undergone CT, vascular imaging and echocardiogram not showing a high-risk large artery or maor-risk cardioembolic source of embolism |

**Table S2: Odds of refusal rate by non-acute/outpatient versus acute/emergency department, multivariable logistic regression**

| Odds ratio (95% CI), p-value | | | |
| --- | --- | --- | --- |
| Model 1 (location only) | Model 2 (+gender) | Model 3 (+gender, age) | Model 4 (+gender, age, intervention) |
| 1.50 (0.81 – 2.78), p=0.20 | 1.58 (0.84 – 2.95), p=0.15 | 1.60 (0.84 – 3.01), p=0.15 | 1.43 (0.68 – 2.99), p=0.35 |

**Table S3: Reasons for refusal separated by gender***

| **Reason** | **Female (n, %)** | **Male (n, %)** |
| --- | --- | --- |
| *Concern about receiving study drug/device* | 16 (33) | 9 (21) |
| *Not interested in participating in study* | 9 (19) | 12 (27) |
| *Concern about inability to choose intervention vs. control* | 4 (8) | 8 (18) |
| *Unable to commit to follow-up appointments* | 3 (6) | 8 (18) |
| *Too stressed or busy* | 4 (8) | 0 (0) |
| *Concerns regarding eligibility for travel insurance* | 2 (4) | 1 (2) |
| *Indecision about participation* | 0 (0) | 2 (5) |
| *Family refusal on patient’s behalf* | 2 (4) | 0 (0) |
| *Multiple medical comorbidities* | 0 (0) | 1 (2) |
| *In denial about medical condition* | 1 (2) | 0 (0) |
| *Unknown* | 7 (15) | 3 (7) |
| *Total* | 48 | 44 |

**Not powered appropriately for meaningful statistical analysis*
